# Supplementary material for: Association of symptoms at heart failure diagnosis with hospitalisation and mortality at 6 and 12 months: a retrospective cohort study using UK primary care health records
Source: BMJ Open. 2026 Feb 12;16(2):e107490. doi: 10.1136/bmjopen-2025-107490 (PMC12911825; doi:10.1136/bmjopen-2025-107490)
Supplement: Supplementary file 2 [file bmjopen-2025-107490.reviewer_comments.pdf]

PEER REVIEW HISTORY

BMJ Open publishes all reviews undertaken for accepted manuscripts. Reviewers are asked to complete a checklist review form and are provided with free text boxes to elaborate on their assessment. These free text comments are reproduced below.

ARTICLE DETAILS

Title (Provisional)

Association of symptoms at heart failure diagnosis and 6 and 12 month post-diagnosis with hospitalisation and mortality: a retrospective cohort study using UK primary care healthcare records

Authors

Ali, Mohammad Rizwan; Lam, Carolyn S. P.; Stromberg, Anna; Hand, Simon PP; Booth, Sarah; Zaccardi, Francesco; McCann, Gerry P; Khunti, Kamlesh; Lawson, Claire A

VERSION 1 - REVIEW

**Reviewer**	**1**
**Name**	**Kelson, Zoe**
**Affiliation**	**University of Exeter, Mathematics**
**Date**	**05-Sep-2025**
**COI**	**None**

This study investigated symptoms reported before and after heart failure (HF) diagnosis and their associations with 3-month hospitalisation and mortality.

Reviewer comments:

"Primary care database (CPRD) linked to hospital and mortality data (1998–2020)...

Among 86,882 HF patients (62,742 and 54,555 surviving to 6 and 12 months, respectively)" [Abstract]

and

"We used the Clinical Practice Research Datalink (CPRD), an anonymised electronic primary database covering over 11.3 million patients.24 This database is an internationally recognised population level database, representative of the general population in terms of age, sex, and ethnicity24 and includes information on sociodemographic, clinical, and lifestyle factors as well as laboratory and prescription data. CPRD uses a representative sample of general practices in the UK and is linked to national datasets including Hospital

Episode Statistics (HES) and Office of National Statistics (ONS), providing hospital and mortality data, respectively. "

The authors do well to utilise a wealth of representative data in this study.

"Based on existing evidence,27 the covariates included in our analysis encompassed sociodemographic variables (age, sex, ethnicity, and socioeconomic status), lifestyle factors (body mass index (BMI), alcohol consumption, and smoking status), drug prescriptions (beta blockers, renin angiotensin system inhibitors (angiotensin-converting enzyme (ACE) inhibitors or angiotensin receptor blockers (ARB)), angiotensin receptor neprilysin inhibitors (ARNI), alpha blockers, sodium-glucose cotransporter (SGLT) 2 inhibitors, aspirin, statins, sodiumglucose cotransporter (SGLT) 2 inhibitors, and loop diuretics) and physiological data (systolic blood pressure, haemoglobin levels, cholesterol levels, and estimated glomerular filtration rate (eGFR)). Additionally, we accounted for several comorbidities, including hypertension, diabetes, ischaemic heart disease (IHD), myocarditis, cerebrovascular accident (stroke), atrial fibrillation (AF), asthma, cancer, cardiomyopathy, chronic obstructive pulmonary disease (COPD), chronic kidney disease (CKD), osteoarthritis (OA), rheumatoid arthritis (RA), dementia, and depression. As a surrogate measure for heart failure (HF) severity, we utilised hospitalisation history (within one month; one to three months; or three to 12 months prior to the landmark point)."

The authors satisfactorily specify how much data is missing in the Tables.

"Landmark analysis using Royston-Parmar survival models at baseline (diagnosis), 6- and 12 months post-diagnosis" [Abstract]

and

"at each landmark point, Royston Parmar flexible parametric survival models30 were used to estimate the association between each symptom and 3-month first all-cause and cause specific hospitalisation and mortality, using the “stpm2” command in Stata 17 (StataCorp, College Station, TX, USA)"

and

"Multicollinearity between all variables in the model was assessed and linearity between each continuous variable and outcome was determined using likelihood ratio tests"

and

"Unadjusted hazard ratios with 95% confidence intervals were estimated followed by adjustment of each symptom by all covariates"

Appropriate analytical methods have been applied by the authors.

"Assuming a missing at random mechanism, multiple imputation using chained equations was performed to impute socioeconomic status, ethnicity, and physiological data (missingness information is reported in Table 1), generating 10 imputed datasets with the results combined using Rubin’s rules"

and

"A sensitivity analysis was undertaken using complete case analysis "

The authors apply a rigorous approach to handling missing data.

"Akaike and Bayesian Information Criteria (AIC and BIC), comparing models with different transformations (a quadratic extension or restricted cubic splines with three degrees of freedom)"

Model performance metrics have been satisfactorily assessed.

The Results, tables and figures, are reported accurately and concisely.

"Key clinical markers of heart failure severity (e.g., ejection fraction, NYHA class, natriuretic peptides) were not available, which may limit the assessment of symptom relevance across different HF phenotypes." [Strengths and Limitations]

and

"However, several limitations need to be acknowledged. Although there is some evidence of large population databases in primary care recording symptoms,49 these may be incomplete. However, under reporting would likely bias the associations towards the null value, leading to underestimated associations in this study. We did not have access to HF phenotype information and further work is needed to ascertain whether symptom associations differ by phenotype. Likewise, we did not have HF severity measures such as ejection fraction, NYHA, or natriuretic peptides. Moreover, we chose the landmarks a priori for clinical reasons. Shorter, longer, or more frequent landmark times may provide better data on important timepoints for symptoms and requires further research"

A discussion on the study limitations is provided by the authors.

Thanks for providing a copy of the STROBE checklist.

**Reviewer**	**2**
**Name**	**Bestetti, Reinaldo**
**Affiliation**	**University of Sao Paulo Faculty of Medicine of Ribeirao Preto, Medicine**
**Date**	**28-Oct-2025**
**COI**	**None**

In this paper, the authors sought to determine predictors of hospitalization and mortality in patients with heart failure (HF) followed in primary care. To achieve this, they used a landmark analysis to assess the predictive value of symptoms observed at baseline (prior to diagnosis), and at 6 and 12 months post-diagnosis, for all-cause and cause-specific hospitalisations and all-cause mortality occurring within 3 months after each landmark

point. They included 86,882 patients with a first recorded diagnosis of heart failure (HF) in an anonymized national primary care electronic database between January 1998 and March 2020. The authors state that HF diagnoses in primary care records were based on clinically validated terms. Symptoms and signs relevant to HF were identified according to the European Society of Cardiology (ESC) guidelines on heart failure, including shortness of breath, ankle swelling, generalized oedema, fatigue, chest pain, anxiety, and depression. At diagnosis, depression was associated with a higher risk of all-cause hospitalisation, while shortness of breath predicted hospitalisation due to heart failure. At 6 months, depression remained associated with all-cause hospitalisation, and ankle swelling was linked to all-cause mortality. At 12 months, shortness of breath was again associated with heart failure hospitalisation. Major comments: This is an interesting study that highlights the importance of assessing risk factors throughout the syndrome trajectory of patients with HF, rather than only at admission, given the fluctuations in symptoms and signs characteristic of this condition. The large number of patients included in the study, and the fact that the investigation was conducted in a primary care setting, are strengths of the paper. In my view, however, an important weakness of the study is that the diagnosis of HF was based on "clinically validated terms" in primary care records, rather than being confirmed by abnormal electrocardiographic findings, elevated natriuretic peptide levels, or echocardiographic evidence, as recommended by the ESC guidelines. The symptoms and signs predictive of unfavourable outcomes are not specific to patients with HF. Furthermore, the severity of HF was assessed based on hospitalisation history rather than left ventricular ejection fraction. I suggest that the authors address this issue in greater depth in the Discussion section. Minor comments: 1) Minor comments: In Figure 4 (forest plot), depression and shortness of breath appear to be associated with all-cause mortality across all three landmark points. This seems to contrast with what is stated in the Abstract. Please clarify this apparent discrepancy; 2) According to the forest plot in Figure 3, pain and depression were associated with non-CVD hospitalization. This finding deserves comment in the Discussion section, as non-CVD conditions are an important cause of hospitalization and mortality in patients with HF.

VERSION 1 - AUTHOR RESPONSE

Reviewer: 1

Prof. Zoe Kelson, University of Exeter

The authors do well to utilise a wealth of representative data in this study.

The authors satisfactorily specify how much data is missing in the Tables.

Appropriate analytical methods have been applied by the authors.

The authors apply a rigorous approach to handling missing data.

Model performance metrics have been satisfactorily assessed.

A discussion on the study limitations is provided by the authors.

Thanks for providing a copy of the STROBE checklist.

Many thanks for your comments. There was nothing within the feedback that required addressing.

Reviewer: 2
Dr. Reinaldo Bestetti, University of Sao Paulo Faculty of Medicine of Ribeirao Preto
Comments to the Author:

Major comments: This is an interesting study that highlights the importance of assessing risk factors throughout the syndrome trajectory of patients with HF, rather than only at admission, given the fluctuations in symptoms and signs characteristic of this condition. The large number of patients included in the study, and the fact that the investigation was conducted in a primary care setting, are strengths of the paper.
Many thanks.

In my view, however, an important weakness of the study is that the diagnosis of HF was based on "clinically validated terms" in primary care records, rather than being confirmed by abnormal electrocardiographic findings, elevated natriuretic peptide levels, or echocardiographic evidence, as recommended by the ESC guidelines.
Diagnosis follows National Institute for Health and Care Excellence (NICE) which recommends a blood test (BNP) followed by echocardiography. The patients who have the diagnosis coded have done so usually after their BNP or following the results of their echocardiography. The clinically validated terms are those that are inputted by the primary care physician (general practitioner) – it would incredibly unlikely a code would be entered unless the clinician had some certainty that the patient had a diagnosis of HF.

The symptoms and signs predictive of unfavourable outcomes are not specific to patients with HF.
One of the novelties in our study was using non-HF specific symptoms. This was done intentionally. We published a core outcome set of important symptoms in people with a HF diagnosis using a consensus methodology bringing together clinicians and patients to determine which symptoms (HF specific and other symptoms) are important in HF (https://doi.org/10.1093/eurjcn/zvac019). We aimed to investigate how these may be important in prognosis for this study.

Furthermore, the severity of HF was assessed based on hospitalisation history rather than left ventricular ejection fraction. I suggest that the authors address this issue in greater depth in the Discussion section.
We thank the reviewer for this important point. We agree LVEF is an important indicator of clinical severity. However, within the UK, particularly in primary care, echocardiographic data is recorded as free text by the GPs yet it is unavailable for researchers to access. To mitigate against the high level of missingness, we used prior hospitalisation as a proxy measure of disease severity as previous evidence has shown previous HF hospitalisation is one of the strongest predictors of future rehospitalisation and mortality. We have referenced this within the discussion as well – this can be found on Page 15.

Minor comments: 1) In Figure 4 (forest plot), depression and shortness of breath appear to be associated with all-cause mortality across all three landmark points. This seems to contrast with what is stated in the Abstract. Please clarify this apparent discrepancy;
We appreciate the close attention to the data in the abstract and apologise for the lack of clarity. The reviewer is correct that statistically significant associations were observed for SOB and depression. However, we wanted to showcase the symptoms presenting the largest hazard ratios at each specific timepoint. We have modified the abstract to ensure this properly reflects that these were the highest risk associations rather than the only statistically significant results.

2) According to the forest plot in Figure 3, pain and depression were associated with non-CVD hospitalization. This finding deserves comment in the Discussion section, as non-CVD conditions are an important cause of hospitalization and mortality in patients with HF.
Thank you. This is an excellent point. "non-cardiac" symptoms (such as pain and depression) and non-CVD hospitalisation highlights the multimorbidity burden in this population. Depression may drive non-CVD admissions through mechanisms such as reduced adherence to medication and/or poor self-care. We have added this on Page 13/14, in the discussion section.

VERSION 2 - REVIEW

Reviewer 2

Name Bestetti, Reinaldo

Affiliation University of Sao Paulo Faculty of Medicine of Ribeirao Preto, Medicine

Date 22-Jan-2026

COI

The authors have properly addressed the issues.
